# RETRACTED: Alhakamy et al. Optimized Ellagic Acid–Ca Pectinate Floating Beads for Gastroprotection against Indomethacin-Induced Gastric Injury in Rats. *Biomolecules* 2020, *10*, 1006

**DOI:** 10.3390/biom14030351

**Published:** 2024-03-14

**Authors:** Nabil A. Alhakamy, Shaimaa M. Badr-Eldin, Osama A. A. Ahmed, Abdulrahman A. Halwani, Hibah M. Aldawsari, Mohamed A. El-Moselhy, Aliaa Anter, Sara S. Sharkawi, Muhammad H. Sultan, Osama A. A. Madkhali, Muhammed A. Bakhrebah, Mohammad N. Alomary, Wesam H. Abdulaal, Usama A. Fahmy

**Affiliations:** 1 Department of Pharmaceutics, Faculty of Pharmacy, King Abdulaziz University, Jeddah 21589, Saudi Arabia; nalhakamy@kau.edu.sa (N.A.A.); oaahmed@kau.edu.sa (O.A.A.A.); dr.a.a.halawani@gmail.com (A.A.H.); haldosari@kau.edu.sa (H.M.A.); uahmedkauedu.sa@kau.edu.sa (U.A.F.); 2 Advanced Drug Delivery Research Group, Faculty of Pharmacy, King Abdulaziz University, Jeddah 21589, Saudi Arabia; 3 Center of Excellence for Drug Research and Pharmaceutical Industries, King Abdulaziz University, Jeddah 21589, Saudi Arabia; 4 Department of Pharmaceutics and Industrial Pharmacy, Faculty of Pharmacy, Cairo University, Cairo 12613, Egypt; 5 Department of Clinical Pharmacy and Pharmacology, Ibn Sina National College for Medical Studies, Jeddah 22413, Saudi Arabia; m_moselhy64@yahoo.com; 6 Department of Pharmacology and Toxicology, Faculty of Pharmacy, Minia University, Minia 61519, Egypt; aliaafath@yahoo.com (A.A.); sara_shaaban82@yahoo.com (S.S.S.); 7 Department of Pharmaceutics, College of Pharmacy, Jazan University, Jazan 45142, Saudi Arabia; mhsultan@jazanu.edu.sa (M.H.S.); omadkhali@jazanu.edu.sa (O.A.A.M.); 8 Life Science and Environment Research Institute, King Abdulaziz City for Science and Technology (KACST), Riyadh 12354, Saudi Arabia; mbakhrbh@kacst.edu.sa (M.A.B.); malomary@kacst.edu.sa (M.N.A.); 9 Department of Biochemistry, Cancer Metabolism and Epigenetic Unit, Faculty of Science, King Abdulaziz University, Jeddah 21589, Saudi Arabia; whabdulaal@kau.edu.sa

The journal retracts the article, “Optimized Ellagic Acid–Ca Pectinate Floating Beads for Gastroprotection against Indomethacin-Induced Gastric Injury in Rats” [1], cited above.

Following publication, concerns were brought to the attention of the Publisher regarding the duplication of images across other publications [2,3], representing different experimental conditions.

Adhering to our complaints procedure, an investigation was conducted by the Editorial Office and Editorial Board that confirmed that Figure 10B from this article [1] overlaps with Figure 7B of the publication [2] and Figure 4B of the publication [3].

While the authors fully cooperated with the Editorial Office during the investigation, they were unable to satisfactorily explain the overlap of figures presenting different experimental conditions and meet the required quality standards of raw images in order to consider a correction as per the journals original image requirements policy (https://www.mdpi.com/journal/biomolecules/instructions#oriimages, accessed on 6 February 2024). As a result, the Editorial Board and Editor-in-Chief were unable to confirm the reliability of the findings and subsequently decided to retract this paper as per MDPI’s retraction policy (https://www.mdpi.com/ethics#_bookmark30, accessed on 6 February 2024).

An Editorial Board Member and the journal Editor-in-Chief were involved, and since they were unable to confirm the reliability of the findings, it was decided to retract the paper.

This retraction was approved by the Editor-in-Chief of the journal *Biomolecules*.

The authors did not agree to this retraction.
